# Age and Season Effect the Timing of Adult Worker Honeybee Infection by *Nosema ceranae*

**DOI:** 10.3389/fcimb.2021.823050

**Published:** 2022-01-28

**Authors:** Clara Jabal-Uriel, Verónica N. Albarracín, Joaquín Calatayud, Mariano Higes, Raquel Martín-Hernández

**Affiliations:** ^1^ Laboratorio de Patología Apícola, Centro de Investigación Apícola y Agroambiental (CIAPA), Instituto Regional de Investigación y Desarrollo Agroalimentario y Forestal (IRIAF), Consejería de Agricultura de la Junta de Comunidades de Castilla-La Mancha, Marchamalo, Spain; ^2^ Facultad de Agronomía y Zootecnia de la Universidad Nacional de Tucumán, Tucumán, Argentina; ^3^ Departamento de Biología, Geología, Física y Química inorgánica, Universidad Rey Juan Carlos, Madrid, Spain; ^4^ Instituto de Recursos Humanos para la Ciencia y la Tecnología (INCRECYT – ESF/EC-FSE), Fundación Parque Científico y Tecnológico de Castilla – La Mancha, Albacete, Spain

**Keywords:** Microsporidia, *Nosema ceranae*, *Apis mellifera*, honeybee, natural infection, age of infection

## Abstract

The microsporidia *Nosema ceranae* is an intracellular parasite of honeybees’ midgut, highly prevalent in *Apis mellifera* colonies for which important epidemiological information is still unknown. Our research aimed at understanding how age and season influence the onset of infection in honeybees and its development in the colony environment. Adult worker honeybees of less than 24h were marked and introduced into 6 different colonies in assays carried out in spring and autumn. Bees of known age were individually analyzed by PCR for *Nosema* spp. infection and those resulting positive were studied to determine the load by Real Time-qPCR. The age of onset and development of infection in each season was studied on a total of 2401 bees and the probability and the load of infection for both periods was established with two statistical models. First *N. ceranae* infected honeybees were detected at day 5 post emergence (p.e.; spring) and at day 4 p.e. (autumn) and in-hive prevalence increased from that point onwards, reaching the highest mean infection on day 18 p.e. (spring). The probability of infection increased significantly with age in both periods although the age variable better correlated in spring. The *N. ceranae* load tended to increase with age in both periods, although the age-load relationship was clearer in spring than in autumn. Therefore, age and season play an important role on the probability and the development of *N. ceranae* infection in honeybees, bringing important information to understand how it spreads within a colony.

## Introduction

The European honeybee, *Apis mellifera*, currently faces many stressors that affect colony viability such as pesticides, climate change, loss of biodiversity, pests and pathogens (vanEngelsdorp and Meixner, 2010). Among the latter, the microsporidia *Nosema ceranae* has a detrimental impact on infected colonies (Higes et al., 2008; Emsen et al., 2020). Initially described in the Asian bee *Apis cerana* (Fries et al., 1996), *N. ceranae* was first reported in 2006 in the European honeybee *A. mellifera* in Europe (Higes et al., 2006) and one year later in Asia (Huang et al., 2007). It has since been reported in other parts of the globe (Klee et al., 2007; Chen et al., 2008; Williams et al., 2008; Giersch et al., 2009), becoming a very widespread and prevalent parasite of honeybees.

*N. ceranae*, which has been proposed to be reclassified as *Vairimorpha ceranae* (Tokarev et al., 2020) even though we maintain the original name, is an obligate intracellular parasite that is acquired by ingesting spores from contaminated food or water, during cleaning duties or through trophallaxis (reviewed in Martín-Hernández et al., 2018). After intake, the spores reach the ventriculum where they extrude the polar filament to introduce the sporoplasm into the bees’ epithelial cells in which the parasite completes its cell cycle and reproduces, extending infection throughout the organ and producing severe tissue damage (Higes et al., 2007; García-Palencia et al., 2010; Maiolino et al., 2014; Panek et al., 2018).

At the individual level, *N. ceranae* infection induces immune modulation (Antúnez et al., 2009; Li et al., 2018), it alters polyethism, promoting infected bees to forage prematurely (Dussaubat et al., 2013; Lecocq et al., 2016) and shortening their lifespan (Higes et al., 2007; Goblirsch et al., 2013). At the colony level, infection reduces colony strength, i. e. population size and honey production (Higes et al., 2010; Botías et al., 2013; Bravo et al., 2014; Simeunovic et al., 2014), that finally can lead to colony collapse (Higes et al., 2008; Higes et al., 2010; Villa et al., 2013; Bekele et al., 2015; Adjlane and Haddad, 2016).

This microsporidia parasitizes colonies all year round, although the levels of infection fluctuate across seasons (Higes et al., 2008; Gisder et al., 2010; Copley et al., 2012; Traver et al., 2012; Gisder et al., 2017; Emsen et al., 2020). While all honeybee castes (queen, drones, workers) can be infected (Higes et al., 2009; Traver and Fell, 2011; Martín-Hernández et al., 2012), the prevalence differs among them, being drones and workers the most infected (Traver and Fell, 2011; Martín-Hernández et al., 2012).

In terms of worker bees, *N. ceranae* parasitize all ages even though the prevalence of infection varies depending on age (Smart and Sheppard, 2012; Jack et al., 2016; Li et al., 2017). In fact, infection is usually more prevalent in foragers than in younger bees that perform in-hive duties (Smart and Sheppard, 2012; Botías et al., 2013; Jack et al., 2016). Thus, the structure of the colony might play an important role in the dynamics of pathogen transmission as the network in which the colony is organized shields younger bees from infection, which has been proposed as a system of organizational immunity (Naug and Smith, 2007; Baracchi and Cini, 2014). Nevertheless, nurse bees are also susceptible to infection (Smart and Sheppard, 2012; Jack et al., 2016) and indeed, *N. ceranae* appears to be capable of infecting larvae (Eiri et al., 2015; Benvau and Nieh, 2017) even under natural conditions (Urbieta-Magro et al., 2019a). However, there is still little knowledge regarding the age at which honeybees begin to be infected by *N. ceranae* in natural conditions, as the information available so far is limited to groups of bees of the same age, such as recently emerged bees, nurses or foragers (Smart and Sheppard, 2012; Jack et al., 2016; Li et al., 2017). Therefore, the objectives of this work were to determine at what age honeybees are infected by *N. ceranae* under field conditions and how age and season influence the development of such infections as this information could help to understand the epidemiology of the infection within the colony.

## Material and Methods

### Experimental Design

**Figure 1:**
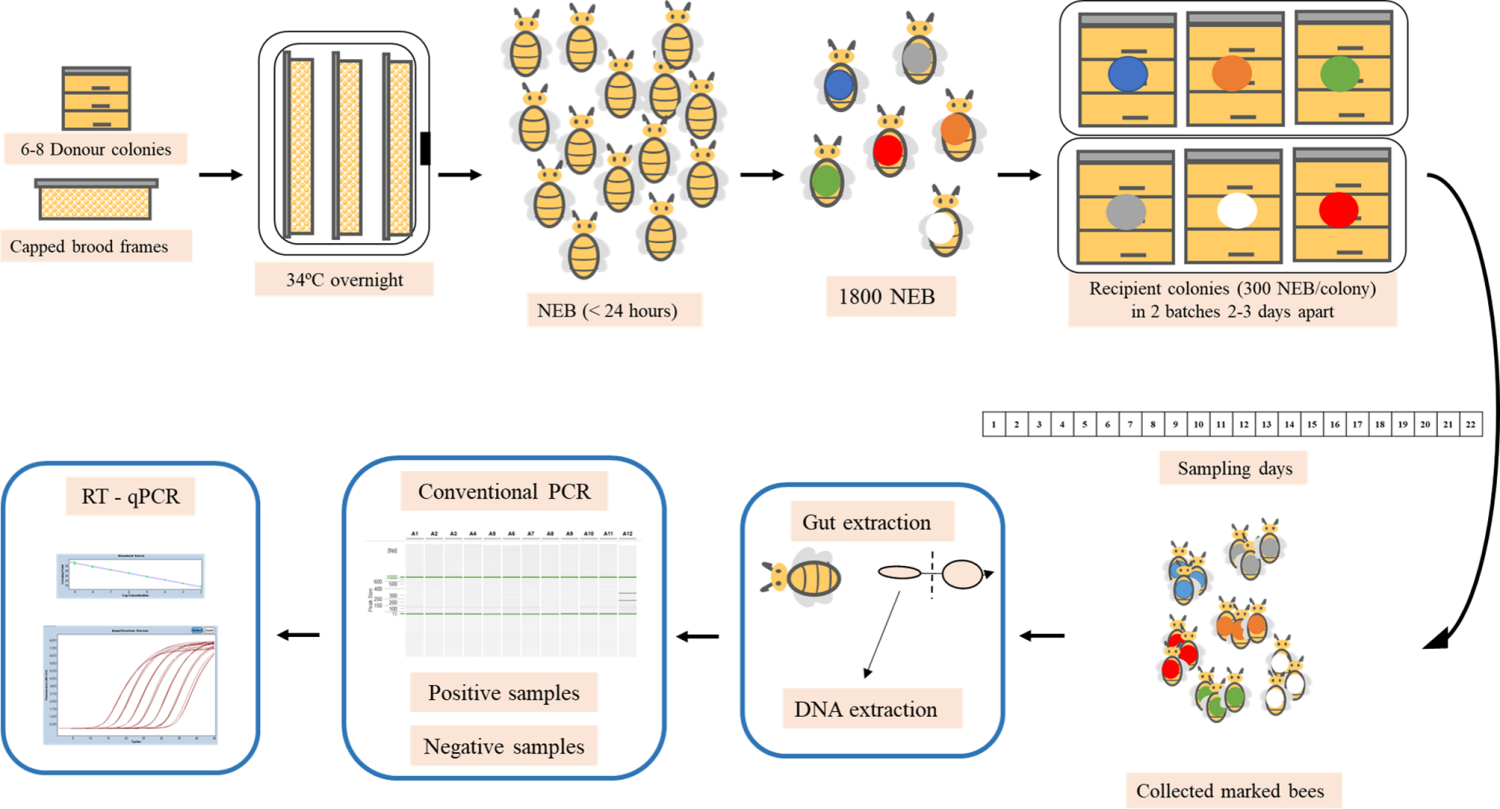
Experimental design of spring and autumn trials. Capped brood frames were taken from a donor apiary and placed overnight in an incubator and the following day, the newly emerged bees (NEB) were marked and placed in the selected recipient colonies. Marked bees were collected every day and processed in the laboratory. The midgut was removed from each bee and molecular analysis were carried out to detect *N. ceranae* infection and whenever the samples were positive, RT-qPCR was conducted to quantify the parasitic load.

This study was carried out in late spring (May-June 2019) and autumn (October 2019) on experimental colonies located at the Centro de Investigación Apícola y Agroambiental (CIAPA), in Marchamalo, Spain (40° 40´55,77” N; 3° 12´ 32,72” W) and it is shown in the **Figure 1**. Meteorological conditions during the experiment can been seen in **Figure S1**.

### Spring Experiment

This assay was performed during the end of May and the first fortnight of June.

To avoid genetic homogeneity, eight capped worker brood combs were taken from 8 A*. mellifera iberiensis* colonies located at an experimental apiary 16 km away from the CIAPA the day before the experiment started. Those colonies had been previously tested and they were negative or had a very low prevalence of *N. ceranae* infection, and they did not show any clinical signs of other diseases. The combs were immediately moved to the laboratory, removing all the remaining adult bees on them, and placed in an incubator (Memmert^®^ IPP 500) at 34 (± 1) °C overnight.

The next day (day 0), the newly emerged adult bees, all of them of less than 24h old, were allotted in three groups of approximately 300 individuals each and marked with an assigned color of enamel paint on the thorax (Posca PC-5M, Mitsubishi Pencil Co.) before they were introduced into three recipient colonies naturally infected with *N. ceranae* (300 marked bees per colony). In addition, 15 newly emerged bees were collected to check for the absence of *N. ceranae* and they were frozen at -20°C until analysis. To make it easier for the new bees to be accepted in the recipient colonies, sugar syrup was sprayed upon them prior to their release. The whole procedure was repeated 3 days later and again, 300 marked bees of each group were released into another three different *N. ceranae* infected colonies. Therefore, a total of 1800 one-day-old marked worker bees were introduced into six recipient colonies. In this way, we had 2 groups of 3 hives, overlapping each other so we were able to collect daily data from at least 3 colonies for the whole studied period (in order to be able to collect samples during working days). During 21 days, 15 marked bees were collected manually every morning, beginning at the same time, from each of the six colonies starting the day after introduction (day 1) and they were taken to the laboratory for analysis.

The recipient colonies were analyzed to determine the basal level of infection. To do this, prior to the introduction of newly emerged bees on day 0, adult worker bees from those recipient colonies were collected after brushing a comb with no brood. From each colony, 25 bees were analyzed individually (honeybee per honeybee) to determine how many of them were infected by *Nosema* spp.

### Autumn Experiment

This assay was carried out in October following the same experimental design as described above. In this case, 6 capped brood combs were taken from 6 colonies in the same donor apiary to avoid genetic homogeneity, taken to the lab and maintained under the same conditions. The newly emerged bees were marked as indicated above, again in two different batches, this time separated by 2 days. In this case, the absence of infection was determined in 15 newly emerged bees sampled each day of marking and release (day 0), such that 30 bees were analyzed in total. Regarding the recipient colonies, three colonies used for the spring trial remained alive and they were used again in this assay. The other three colonies died (colonies 1, 3 and 6) and they were replaced by three other naturally infected colonies (colonies 7, 8 and 9). During this trial, the marked bees were collected over 22 days as indicated and *Nosema* spp. infection of all the recipient colonies was again determined as described above (25 worker bees/colony) at the day of introduction.

### Molecular Detection of *Nosema* spp.

Once the marked bees were collected each morning, they were transported to the laboratory and processed immediately. The gut of each honeybee was carefully removed by pulling the last segment of the abdomen with sterile tweezers in a laminar flow cabinet (Telstar AV–30/70). Each gut was cut with sterile dissection scissors, separating the ventriculum and rectum by a cut at half of the ileum. The first part, containing the ventriculum and half of the ileum, was used in this study. Each sample was placed in one well of a 96-well plate (Qiagen^®^) containing 250 µL of sterile PBS buffer and four 2 mm glass beads (Sigma^®^) for homogenization. The tissues were homogenized for 2 minutes at 30 Hz (TyssueLyser II, QIAgen^®^). A well was left with PBS buffer and the reagents alone as a control for DNA extraction between the bees from different colonies and days.

In both the spring and autumn trials, the presence of *Nosema* spp. was checked in newly emerged bees collected on day 0 and those adult bees collected to determine the basal infection in the recipient colonies, processing the bees individually. As such, the full abdomen of each honeybee was placed in a well of a 96-well plate (Qiagen ^®^) containing 250 µL of nuclease-free water (Sigma^®^) and four 2 mm glass beads (Sigma^®^), and homogenized as indicated above.

DNA was extracted as described previously (Urbieta-Magro et al., 2019b) transferring 50 µL of the homogenate to 50 µL of a Tris-HCl lysis solution (10 nM Tris-HCl [pH 8.0], 1 nM EDTA - TE). All the DNA samples obtained were kept frozen (-20°C) until further analysis. To assess the presence of *Nosema* spp., conventional triplex PCR was performed on all the samples using gelified plates (BioTools^®^) (Martín-Hernández et al., 2012) in a Mastercycler^®^ ep gradient S (Eppendorf) and the resulting amplicons were analyzed in a QIAxcel Advanced System (Qiagen^®^) following the protocol described elsewhere (Urbieta-Magro et al., 2019b), including an internal control of *A. mellifera* DNA. In addition, the aforementioned DNA extraction controls, non-template controls (NTCs), and a positive control of *N. ceranae* and *N. apis* were included in the reaction plates.

### Quantification of the *N. ceranae* Load Per Bee

The bees of known age (marked bees) that were detected positive for *Nosema* spp. were afterwards analyzed by real time quantitative-PCR (RT-qPCR) to detect the *polar tubule protein 3* (*PTP3*) gene and determine the *N. ceranae* load per honeybee using the primers and conditions as previously described in Urbieta-Magro et al. (2019b). This was performed in 384-well plates in a Roche LightCycler^®^ 480 thermocycler in a final reaction volume of 20 µL. The amplification cycles were analyzed with the LightCycler^®^ 480 software v1.5.1 (Roche Diagnostics GmbH, Basel, Switzerland) and the crossing point (Cp) was recorded in all the samples using the second maximum derivative method for its calculation based on the standard curve. For each sample, there were two replicates in the same kinetic qPCR run (intra-assay variation), and negative and *N. ceranae* positive controls were analyzed in parallel. The parasite load (pg/µL) was quantified in all samples relative to the specific synthetic oligonucleotides (gBlocks^®^, IDT DNA Technologies, Coralville, Iowa, USA) for the *N. ceranae*-*PTP3* gene fragment (Urbieta-Magro et al., 2019b). The standard curve for quantification was prepared following the manufacturer’s protocol at an initial concentration of 10 ng/μL (in TE) and using serial dilutions up to 1 × 10^−14^ ng/μL. In addition, the GAPDH gene of *A. mellifera* was analysed in those samples to check for DNA integrity using the primers and probe at the same concentration as described in Martín-Hernández et al. (2017), with 5 µL of the DNA template in a final reaction of 20 µL using a Roche LightCycler^®^ 480 thermocycler. All RT-qPCR programmes consisted of an initial 10 min denaturation step at 95°C, and 45 cycles of 10 seconds at 95°C and 30 seconds of annealing at 60 °C, 1 second at 72°C and ended by a cooling step at 40°C for 30 seconds.

### Statistical Analyses

The probability of *Nosema* spp. infection in bees was analyzed using a Generalized Linear Mixed Model fit by maximum likelihood (Laplace approximation) with binomial distribution and logit link function. Hive and sampling date were included as random variables to account for the potential effects of colony idiosyncrasies and daily climatic variations. As fixed variables we included age and its quadratic term, season and the basal infection of the recipient colonies in the moment of introduction. Moreover, an interaction between age and season was also included. Starting from this full model, we followed a backward model selection based on the second order variant of Akaike Information Criteria (AICc). In case two models were equivalent (i. e. ΔAICc ≤ 2) the model with less parameters was chosen, following a rule of parsimony. We calculated the model’s goodness of fit following Nakagawa and Schielzeth (2013) as implemented in the R package MuMIn (Barton, 2020). A Linear Mix Model fit by maximum likelihood was used for the model of *N. ceranae* load. The dependent variable was the log transformed load and the fixed variables were age and season. We included the same fixed and random variables as explained above, using also a backward model selection based on AICc. Calculations were made in R (R 4. 1. 0. http://cran.r-project.org/) using the lme4 package (Bates et al., 2015) to fit mixed models and using the AICcmodavg package (Mazzerolle, 2019) to compute the AICc scores. In addition, the loads were analyzed using SPSS software for Windows (Version 12.0), performing an ANOVA and Games-Howell *post hoc* analysis to correlate the *N. ceranae* loads among the different ages (in days) of the bees during and within both seasons.

## Results

### Infection With *Nosema* spp.

All DNA extraction controls and NTCs did not show any amplification product, indicating the absence of cross-contamination during sample processing and analysis. Significantly, *N. apis* was not detected in any honeybee throughout the study.

None of the 15 newly emerged bees analyzed were infected by *N. ceranae* on the day of introduction (day 0) in spring. However, a single honeybee of the 30 bees analyzed in autumn was positive for *N. ceranae*, which represents a 3.3% of newly emerged bees infected in autumn and a 2.2% among all the newly emerged bees (spring and autumn) analyzed on day 0. The basal infection in the recipient colonies ranged from 8-20% in spring and 4-28% in autumn (**Table 1**).

**Table 1:** Daily percentage (and absolute number) of bees infected per day detected positive for *N. ceranae* infection in each colony during spring and autumn assays.

Colony	Basal infection	Days post – emergence																						Mean % (No.) of infected bees	Marked bees analyzed
		1	2	3	4	5	6	7	8	9	10	11	12	13	14	15	16	17	18	19	20	21	22		
**Spring**																									
**1**	16			0 (0)	0 (0)	33 (5)	20 (3)				47 (7)	40 (6)	47 (7)	40 (6)	47 (7)			60 (9)	77^h^ (10)	83^g^ (10)	93 (14)	80 (12)		47 (96)	205
**2**	8			0 (0)	0 (0)	7 (1)	7 (1)				40 (6)	47 (7)	73 (11)	80 (12)	87 (13)			100 (15)	100 (15)	92^h^ (12)	60^c^ (3)	–		52 (96)	183
**3**	20			0 (0)	0 (0)	7 (1)	0 (0)				20 (3)	40 (6)	27 (4)	20 (3)	53 (8)			67 (10)	85^h^ (11)	80 (12)	67 (10)	87 (13)		39 (81)	208
**4**	12	0 (0)	0 (0)				7 (1)	7 (1)	20 (3)	13 (2)	33 (5)			20 (3)	47 (7)	40^e^ (4)	55^f^ (6)	30^e^ (3)						21 (35)	166
**5**	16	0 (0)	0 (0)				0 (0)	27 (4)	0 (0)	20 (3)	13 (2)			20 (3)	40 (6)	27 (4)	47 (7)	60 (9)						21 (38)	180
**6**	8	0 (0)	0 (0)				7 (1)	20 (3)	7 (1)	33 (5)	33 (5)			31^h^ (4)	67^d^ (4)	100^a^ (1)	–	–						19 (24)	125
Daily Mean % of infection		0	0	0	0	15	7	18	9	22	31	42	49	35	56	34	50	66	88	85	77	83		35	1067
Std. Dev		0	0	0	0	7	6	8	4	10	28	19	22	31	45	9	13	46	36	34	27	25		5	
Sum of bees infected/day		0	0	0	0	7	6	8	4	10	28	19	22	31	45	9	13	46	36	34	27	25		370	
**Autumn**																									
**2**	12	0 (0)	0 (0)			0 (0)	7 (1)	7 (1)	0 (0)	7 (1)			13 (2)	73 (11)	7 (1)	13 (2)	7 (1)			27 (4)	60 (9)	78^i^ (11)	0^b^ (0)	19 (44)	226
**4**	8			0 (0)	7 (1)	13 (2)	7 (1)	7 (1)			20 (3)	13 (2)	0 (0)	0 (0)	13 (2)			7 (1)	13 (2)	40 (6)	20 (3)			12 (24)	210
**5**	28			0 (0)	0 (0)	13 (2)	13 (2)	7 (1)			20 (3)	60 (9)	33 (5)	27 (4)	53 (8)			53 (8)	33 (5)	73 (11)	53 (8)			32 (66)	209
**7**	12	0 (0)	0 (0)			0 (0)	20 (3)	13 (2)	7 (1)	27 (4)			13 (2)	60 (9)	27 (4)	60 (9)	40 (6)			40 (6)	73 (11)	80 (12)	53 (8)	34 (77)	240
**8**	24	0 (0)	0 (0)			0 (0)	0 (0)	0 (0)	13 (2)	0 (0)			7 (1)	40 (6)	13 (2)	7 (1)	73 (11)			33 (5)	73 (11)	50^i^ (7)	7 (1)	20 (47)	239
**9**	4			0 (0)	0 (0)	0 (0)	0 (0)	0 (0)			27 (4)	33 (5)	60 (9)	40 (6)	40 (6)			50^i^ (7)	73 (11)	80 (12)	47 (7)			32 (67)	210
Daily Mean % of infection		0	0	0	2	4	8	6	7	11	22	36	21	40	26	27	40	36	40	49	54	70	28	24	1334
Std. Dev		0	0	0	4	7	7	5	6	11	6	25	20	24	17	24	32	22	29	26	23	35	9	9	
Sum of bees infected/day		0	0	0	1	4	7	5	3	5	10	16	19	36	23	12	18	16	18	44	49	30	9	325	

Fifteen bees were analysed from each colony and day unless specified with a letter in superscript (No. of bees per superscript: ^a^1, ^b^2, ^c^5, ^d^6, ^e^10, ^f^11, ^g^12, ^h^13, ^i^14). Basal infection: Percentage of worker bees infected on each of the recipient colonies at the moment of introduction. %: percentage; Std. Dev.: Standard deviation. – Marked bees not found.

**Figure 2:**
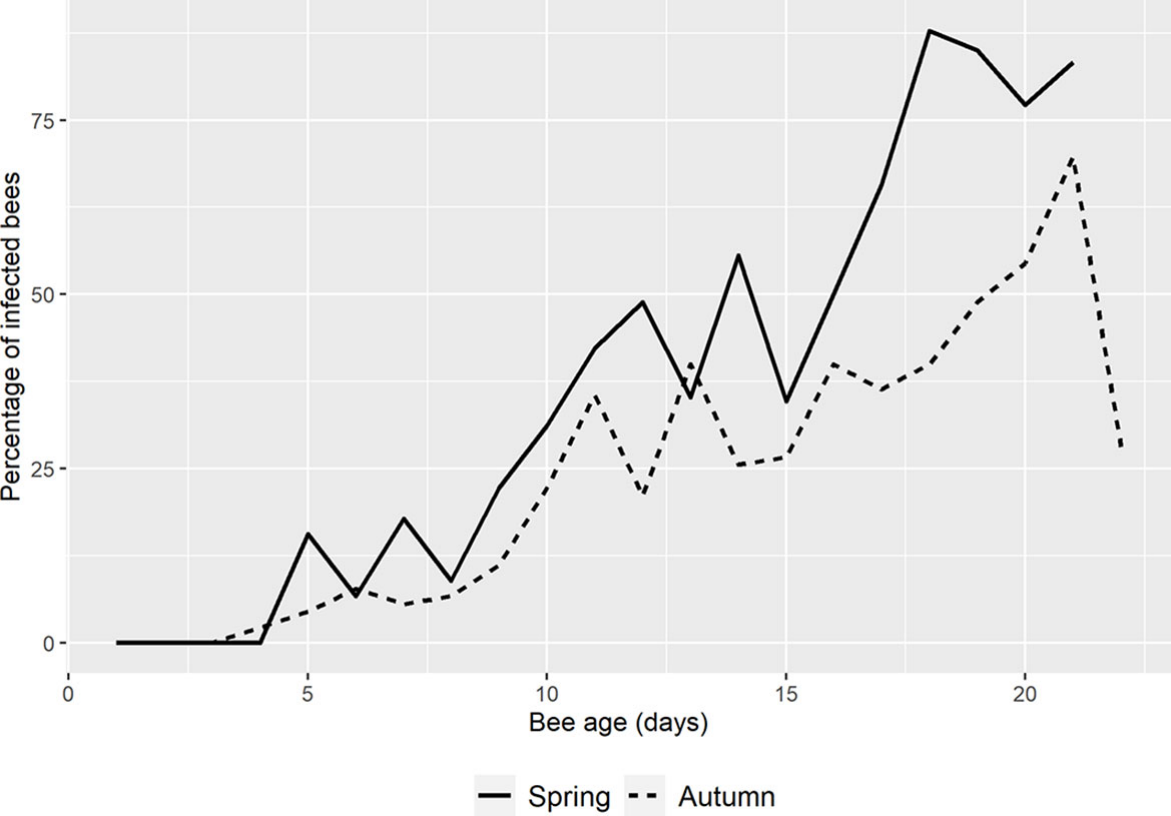
Mean percentage of *N. ceranae* in spring and autumn. The percentage in spring (solid line) starts one day later than in autumn (dotted line) and it remains higher except on days 6 and 13 p.e. On the last day of autumn, the percentage of infection drops dramatically.

A total of 2401 bees (1067 in spring and 1334 in autumn) of known age (from day 1 until day 22 post-emergence - p.e.) were collected and analyzed in this study. Of those collected in spring, 35% were positive for *N. ceranae* when analyzed by conventional triplex-PCR whereas 24% of those collected in autumn were positive (**Table 1** and **Figure 2**).

In spring the first *N. ceranae* infected bees were detected at day 5 p.e. in the three colonies analyzed (33%, 7%, 7% respectively; **Table 1** and **Figure S2**). In autumn, a single honeybee was positive in one colony on day 4 p.e. (**Table 1**) and 4 infected bees were detected in two colonies on the following day. From that point onwards, infection was detected in most of the colonies in spring and autumn, and the percentage of bees infected by *N. ceranae* generally increased (**Figure 2**, **Figure S2**). In spring, the highest mean infection was observed on day 18 p.e. and it remained above 75% until day 21 p.e. This was the general trend seen in all the colonies except for colonies 2 and 4, where a decrease was observed at the end of the study period (**Table 1**). In fact, this latter colony was that with the lowest infection in spring (max. 55%). Two colonies reached 100% infection, colony 2 on days 17 and 18 p.e., and colony 6 on day 15 p.e. However, only one honeybee was found in this latter colony that was positive on day 15, which was the actually last day when any marked honeybee was observed.

In autumn, the mean infection increased until day 21 p.e. when it reached its highest peak (70%), which was followed by a fall to 28% on day 22 p.e. (**Figure 2**). In general, the mean relative infection was lower in this season than in spring, a trend observed in all the colonies, and no colony reached 100% infection. Moreover, colony 4 was again the colony with the lowest level of infection (max. 40%).

**Figure 3:**
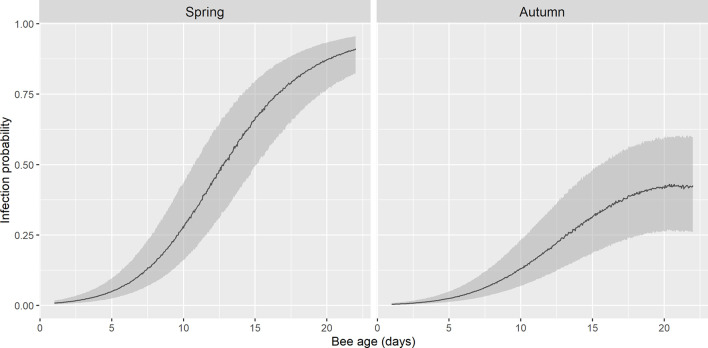
Fitted probability of infection by *N. ceranae* in spring and autumn by GLMM. There is an exponential growth of infection in spring until the onset of foraging, whereas in autumn this probability remains lower. The shadow area represents 95% confidence interval.

The basal infection by *N. ceranae* of the recipient colonies did not have a significant effect on the probability of infection of the marked bees (p>0.05), therefore this parameter was not selected in the best model (**Table 2**). Indeed, the earliest detection of *N. ceranae* infection was seen in colony 4, associated with 8% basal infection. According to the observations, the best model based on the AICc included the age of the bee, its quadratic term and the interaction of both with the season (**Table 2** and **Table S1**). The overall model fit was R2m = 0.47; R2c = 0.53. On average, the probability of infection was higher in spring (least square mean = 0.364, SE = 0.564) than in autumn (mean = 0.170; SE = 0.564). Moreover, although the probability of infection increased significantly with age in both periods, the effect of age was stronger in spring than in autumn (**Figure 3**).

**Table 2:** Results of model selection based on AICc for the probability of infection by *N. ceranae* and the *N. ceranae* load in adult bees.

Model	Variables	AICc	Delta AICc	wAIC
Infection	Age * Season + Age^2^ * Season + Basal Infection	2140.17	0.00	0.53
	Age * Season + Age^2^ * Season	2141.50	1.33	0.27
	Age + Age^2^ + Season + Basal Infection	2142.35	2.18	0.18
	Age * Season + Basal Infection	2146.82	6.65	0.02
Load	Age * Season	3275.71	0.00	0.68
	Age * Season + Basal Infection	3277.67	1.95	0.26
	Age * Season + Age^2^ * Season + Basal Infection	3280.71	5.00	0.06
	Age + Season + Basal Infection	3284.51	8.79	0.01

Age: Days post-emergence of the bees. Season: Spring/Autumn. Basal Infection: % infection of recipient colonies at the moment of introduction.

* denotes the interaction between explanatory variables.

### *N. ceranae* Load in the Infected Bees

This parameter was analyzed in 666 bees which have been determined positive by conventional PCR, as out of the 695 bees that were positive for *N. ceranae* infection, 6 wells broke and the DNA was poorly preserved in 23 samples (GAPDH not detected), excluding these from the subsequent analysis. All the extraction controls and NTCs gave a negative result, indicating the absence of any contamination or non-specific DNA amplification.

**Figure 4:**
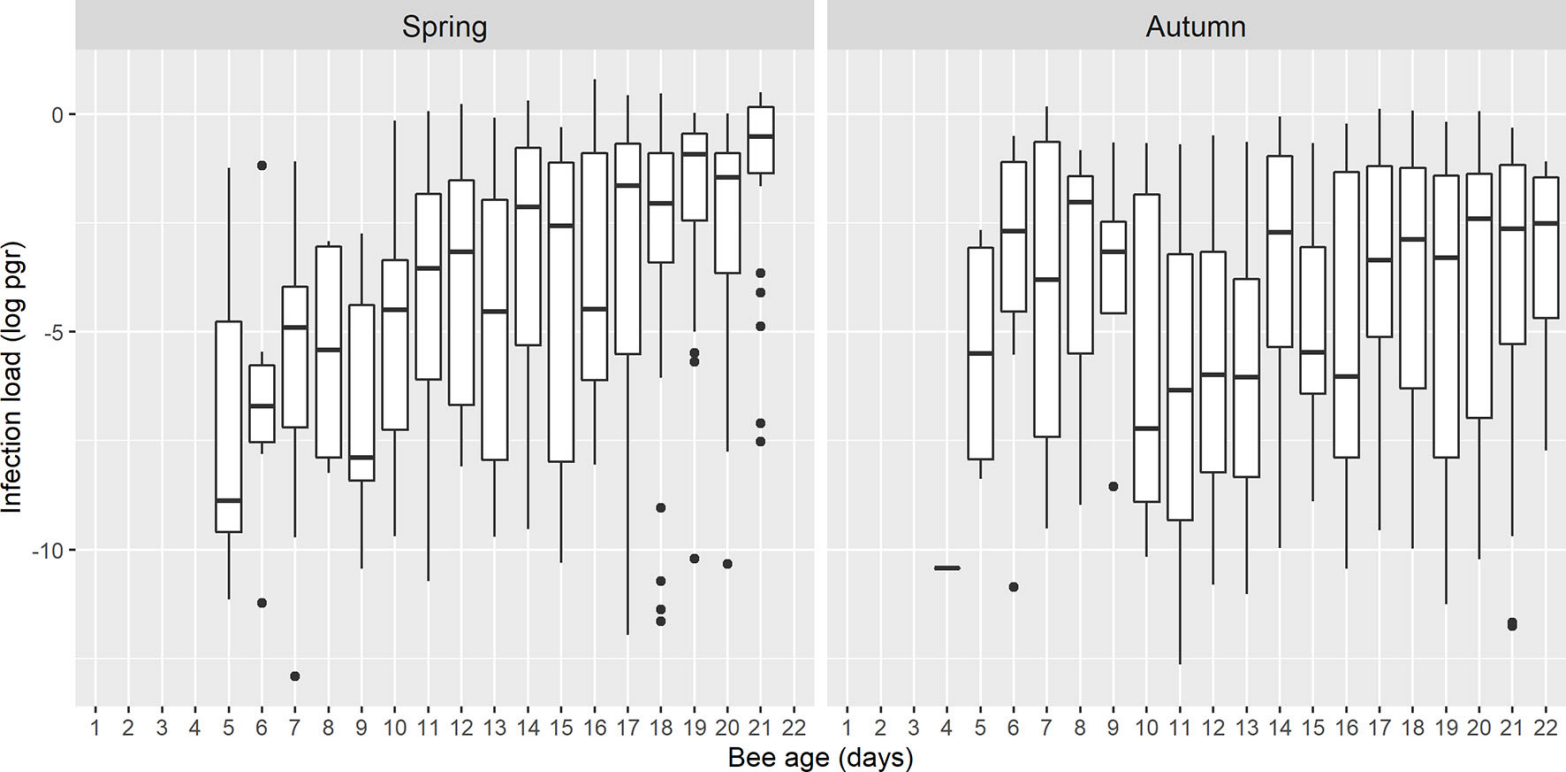
Boxplots showing the *N. ceranae*-*PTP3* load of picograms in logarithmic scale (log pgr) of the infected bees on each day in spring and autumn. The line represents the median while the box represents the 50% of the observations and the whiskers reach the 1.5 x interquartile range. Outliers are also shown as dots.

The load of *N. ceranae*-*PTP3* gene was calculated using a standard curve and the limit of quantification (LOQ) was established at 2.5 × 10^-6^ pg/µL. After analysis, 30 samples were found to be below the LOQ and therefore, they were also removed from the subsequent calculations. As such, the *N. ceranae* load was calculated for 357 bees collected in spring and 279 bees collected in autumn (636 bees in total). The average concentration of the *N. ceranae*-*PTP3* DNA (pg/µL) was assessed in both seasons (**Figure 4**) and no significant differences were evident when the load on a given day was compared between them, except between the bees at 21 days p.e., that had higher load in spring (Games-Howell, p < 0.05). This specific day had a significantly higher load than other days in spring but also, to some other of autumn. The *N. ceranae*-*PTP3* load in autumn did not show significant differences between the days of sampling (Games-Howell; p > 0.05), whereas in spring the bees from days 8 and 9 p.e. had significantly lower loads than those bees from days 14, 17, 19, 20 and 21 p.e. (Games-Howell, p < 0.05).

**Figure 5:**
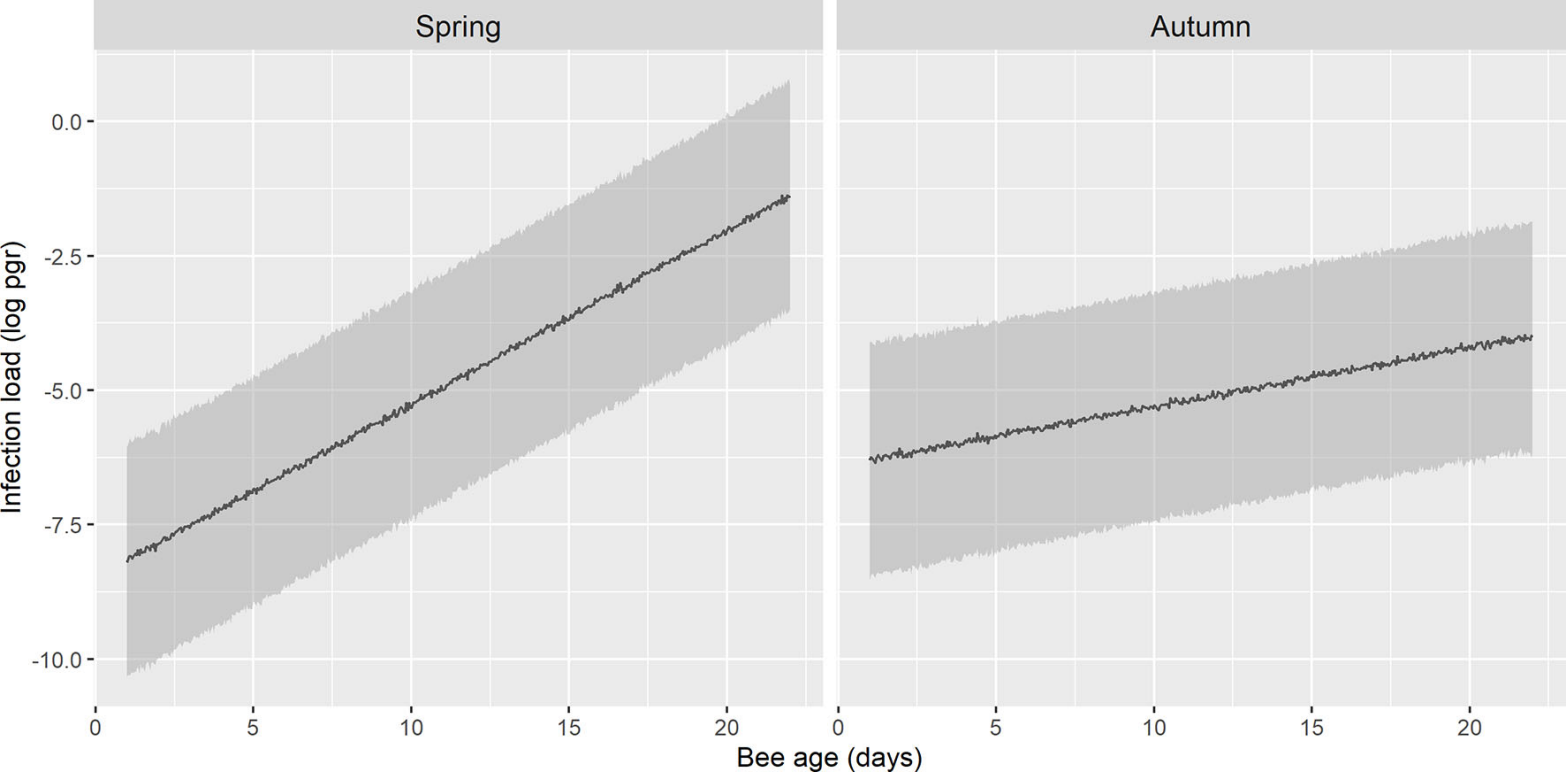
Prediction of *N. ceranae-PTP3* load of picograms in logarithmic scale (log pgr) in infected bees in spring and autumn. The load in spring is lower during the initial days and it increases steeply, whereas in autumn the trend remains flatter across the days.

The best model to explain the *N. ceranae* load in the bees included the age of the honeybee interacting with season (**Table 2** and **Table S2**). The overall model fit was R2m = 0.12; R2c = 0.16. On average, the load was higher in spring (least square mean = 0.028, SE = 1.48) than in autumn (least square mean = 0.007, SE = 1.52). The *N. ceranae* load in the bees tended to increase with age in both periods, although the age-load relationship was steeper in spring than in autumn when it was almost flat (**Figure 5**).

## Discussion

Honeybee colonies have evolved as high-level units of biological organization, assembling unified societies of lower-level units. In fact, a honeybee colony is considered a single living entity formed by a society of many thousands of individuals that functions as a unified whole or superorganism (Seeley, 2010). These superorganisms have developed social immunity by coordinating behavioral interactions among nest mates, which results in colony-level immune responses (reviewed in Evans and Spivak, 2010). Among these collective defenses, social organization has been proposed to represent a barrier to pathogen transmission (Evans and Spivak, 2010). Indeed, social organization leads to organizational immunity (Naug and Smith, 2007) whereby the nurse bees and larvae are in the more isolated part of the colony to reduce their contact with pathogens.

However, despite this complex colony organization, our data indicate that adult bees begin to be infected by *N. ceranae* as young as 4 (autumn) or 5 days (spring) after their emergence. The number of bees infected increases in a time dependent manner, first moderately until day 9 p.e. and then rising remarkably from this point until the age when bees become foragers. To the best of our knowledge, this is the first report confirming the age of infection of worker bees at a colony level, covering every day from their emergence until the onset of foraging and comparing two different seasons. Other reports investigating groups of bees of a range of ages have confirmed older bees to be those with the higher prevalence of *N. ceranae* infection (Higes et al., 2008; Meana et al., 2010; Smart and Sheppard, 2012; Jack et al., 2016; Li et al., 2017). Our data show that infection at early ages was not anecdotal as a total of 12 bees from 5 colonies were infected by day 5 p.e. Therefore, despite the comb compartmentalization to reduce the overlap of bees of different ages (Naug, 2008; Baracchi and Cini, 2014), our data clearly demonstrate that young bees are exposed to *N. ceranae* spores. In this study, a single newly emerged bee was infected by *N. ceranae* and just in autumn and no other infected-bee was detected until day 4 p.e. Therefore, this low prevalence in newly emerged bees did not have a major influence on the results. In fact, of the 695 positive bees only 15 could have been infected from when they emerged (2.2% infected in day 0). This may not be surprising since *N. ceranae* infected capped brood has been reported in the same location which might acquire the infection during larvae stage, probably due to the food provide by nurse bees (Urbieta-Magro et al., 2019a). Due to the absence of treatments to control *Nosema* infection and the high prevalence of *N. ceranae* in Spain, procurement of *Nosema*-free colonies from which to obtain such a large number of newly emerged bees is currently not possible.

Some differences were found in the development of the infection when compared spring and autumn, which was higher in spring and with an earlier increase in the probability of infection (**Figure 3**). Similar results have been reported previously when comparing bees of unknown age collected in spring, summer and autumn (Mulholland et al., 2012; Emsen et al., 2020). In our study, we focused on spring rather than in summer due to the harsh conditions (high average temperature and low relative humidity in the experimental site, central Spain) during that season since the time required for the collection of samples could influence the viability of colonies during the experiment.

There was considerable variability in the mean *N. ceranae* load per bee, consistent with previous results (Mulholland et al., 2012). In the first days of infection in spring, the *N. ceranae*-*PTP3* load was significantly lower than at the end but in autumn there were no differences over the days studied (**Figure 4**). By contrast, no significant differences were found when comparing bees of the same age between spring and autumn, except on day 21 p.e. The different patterns observed between spring and autumn could be related to colony size and the physiological features of the bees in those seasons. In spring, colonies were larger and all the colonies except one had a shallow super, while on the contrary, the colonies were just the nest in autumn. This might influence the distribution of the honeybee population in the colony and therefore, the possibility of contact among bees of different ages. By contrast, previously reported physiological differences between spring and winter bees might explain the different patterns found here. Winter (or diutinus) bees have swollen hypopharyngeal glands, increased fat body size storing proteins and fat, higher vitellogenin concentration and a considerably extended worker lifespan (Morse, 1985; Ricigliano et al., 2018; Dostálková et al., 2021), and they also display differential microbiota (Kešnerová et al., 2020) and different content of physiologically essential, electrolytic, enzymatic of toxic bioelements (Ptaszyńska et al., 2018). These bees exhibit as well a more intense immune response with stronger expression of antimicrobial genes and more intense antimicrobial activity (Dostálková et al., 2021). This might enhance the bee’s resistance to infection, reducing the number of bees infected compared to spring.

The size of the dataset collected here after analysing more than two thousand bees (from 9 colonies) allowed us to test the effect of age and season on the risk of *N. ceranae* infection (i) and the parasite load (ii). For both models, age was correlated with season, and the effects of age were stronger in spring than in autumn, therefore, an old honeybee in spring has a higher probability of being infected and it will have a higher parasite load (**Figures 3**, **5**), what is in accordance with other studies where prevalence of *N. ceranae* infection was found higher in spring than in autumn (Gisder et al., 2017; Emsen et al., 2020). Regarding the probability of infection by *N. ceranae* (i), our model explains 47% of the cases considering only the variables age and season. Other factors like the colony and the sample date enhanced the model fit (53%) but they were included as random effects as some information that might have helped explain the probability of infection was not available. These factors include the genetic background of the colonies, given that nuclear and mitochondrial variability among *A. mellifera iberiensis* colonies in the same apiary have been published (Henriques et al., 2021b), to some unpredictable daily events (e.g. the time required to sample each colony increased as the number of bees dropped throughout the study, weather conditions, etc.). Similarly, the model of parasite load (ii) explains 12% of the variability with age and season. The high percentage of unexplained variability could be due to factors like individual honeybee genetics, differential microbiota (Engel et al., 2015; reviewed in Zheng et al., 2018), coinfection with other pathogens (Forsgren and Fries, 2010; Costa et al., 2011; Doublet et al., 2015; Zheng et al., 2015; Ptaszyńska et al., 2021) or the nutraceutical properties of food (Nanetti et al., 2021), which might differ among the colonies.

The establishment of infection in very young bees and the different progression of infection between seasons might have important consequences on the posterior development and viability of the colony. Indeed, *N. ceranae* infection plays an important role on honeybee polyethism (Dussaubat et al., 2013; Lecocq et al., 2016; Natsopoulou et al., 2016; Mayack et al., 2021) and colony dynamics (Higes et al., 2008; Goblirsch et al., 2013; Wolf et al., 2016; Biganski et al., 2017).

It would be interesting to further investigate these factors that might influence the probability of infection and parasitic load. As a matter of fact, new tools such SNPs (single nucleotide polymorphism) assays are being developed to improve our understanding of immune gene diversity in honeybees (Henriques et al., 2021a). Both the quality and quantity of the feed the bees receive and the presence of nutraceutical substances that may be present in it could modify the development of the infection. Indeed, high-quality or high-diversity pollen have been reported to increase the survival of *N. ceranae* infected honeybees (Di Pasquale et al., 2013; Ferguson et al., 2018; Castelli et al., 2020) despite that it could increase the parasite load (Jack et al., 2016). Certainly, there are some differences in pollen quality according to season, being generally more abundant and diverse in spring than in autumn. Although we did not collect pollen data in this study, the diversity of plants available for foraging in autumn in the experimental area (Asteraceae and Cruciferae, Dr. González Porto, personal communication) was adequate to cover the needs of the colony and did not require additional supplementation. On the other hand, naturally derived bioactive compounds that are gathered by bees from plants could have beneficial effects on health (nutraceutical effect), as recently shown for *N. ceranae* infection (Nanetti et al., 2021), and they may vary among colonies as foragers select different plants (González-Porto, personal communication). With regard to coinfection with other pathogens, the interaction of *N. ceranae* have has been investigated mainly with *N. apis* (Forsgren and Fries, 2010) and deformed wing virus (Costa et al., 2011; Doublet et al., 2015; Zheng et al., 2015). Hence, exploring the effects of other viruses on *N. ceranae* infection could be of interest.

Finally, the bee’s microbiota might also influence the age of infection or even its development, although this has not yet been well stablished. Newly emerged bees have very few or no gut bacteria but if they are exposed to natural hive conditions, as in this study, a stable community dominated by core microbial species will be established by days 4 to 6 p. e. (Powell et al., 2014; reviewed in Kwong and Moran, 2016). Nevertheless, some non-core bacterial species (Engel et al., 2015; reviewed Zheng et al., 2018) or even variants of core gut symbionts (Rubanov et al., 2019) might be present at variable levels among individuals or found in the colony but not in every individual and the presence of these bacteria even differ between seasons (Kešnerová et al., 2020), possibly explaining the individual variation observed here. Besides that, *N. ceranae* infection modulates the gut microbiota by modifying the relative abundance of some bacterial species. Indeed, positive associations have been reported between this microsporidia and some major bacteria such as *Gilliamella. apicola*, *Snodgrasella alvi* and *Bifidobacteria* spp. (Huang et al., 2018; Rubanov et al., 2019; Zhang et al., 2019; Paris et al., 2020). Indeed, it has been suggested that associations between *N. ceranae* and normal gut microbiota could sustain host survival and benefit the pathogen, as bees with an enhanced natural microbiota have higher parasite loads but higher survival, increasing disease transmission, (Zhang et al., 2021). Therefore, the study of the interaction between the parasite and the microbiota highly deserves future research.

In summary, age and season play an important role on the probability and the dynamics of *N. ceranae* infection in bees. Although some other factors might influence this, as indicated above, this is a good starting point to understand how *N. ceranae* spreads within a colony.

## Data Availability Statement

The raw data supporting the conclusions of this article will be made available by the authors, without undue reservation.

## Author Contributions

The study was designed by RM-H, VA, and MH. The experiments (marking bees, collection of samples from colonies and subsequent molecular analysis) were done by CJ-U and VA. The statistical analysis was performed by JC and CJ-U. RM-H and CJ-U wrote the manuscript, which was revised and approved by all the other authors (VA, JC, and MH).

## Funding

This work was funded by the Consejería de Educación, Cultura y Deportes, of the Junta de Castilla – La Mancha (European Regional development Fund) project No. SBPLY/19/180501/000334. INCRECYT program funded by ESF/EC (Fondo Social Europeo). CJ-U contract funded by the Ministerio de Asuntos Económicos y Transformación Digital (grant no. BES-2017-080176, RTA-2015-0003-CO3-01).

## Conflict of Interest

The authors declare that the research was conducted in the absence of any commercial or financial relationships that could be construed as a potential conflict of interest.

## Publisher’s Note

All claims expressed in this article are solely those of the authors and do not necessarily represent those of their affiliated organizations, or those of the publisher, the editors and the reviewers. Any product that may be evaluated in this article, or claim that may be made by its manufacturer, is not guaranteed or endorsed by the publisher.

## Supplementary Material

Supplementary Figure 1 Average, maximum and minimum Temperature and relative humidity at the apiary site from May to October, 2019. Grey rectangles mark the time of spring and autumn assays.

Supplementary Figure 2 Percentage of N. ceranae infection in each colony during the spring and autumn assays.
