# Supplementary material for: Age and Season Effect the Timing of Adult Worker Honeybee Infection by Nosema ceranae
Source: Front Cell Infect Microbiol. 2022 Jan 28;11:823050. doi: 10.3389/fcimb.2021.823050 (PMC8836290; doi:10.3389/fcimb.2021.823050)

Supplementary Figure 2: Spring experiment. Two groups of three colonies each were sampled from day 1 post emergence (p.e) until day 21 p.e. The graphs show the percentage of bees infected by *N. ceranae* per day of sampling.

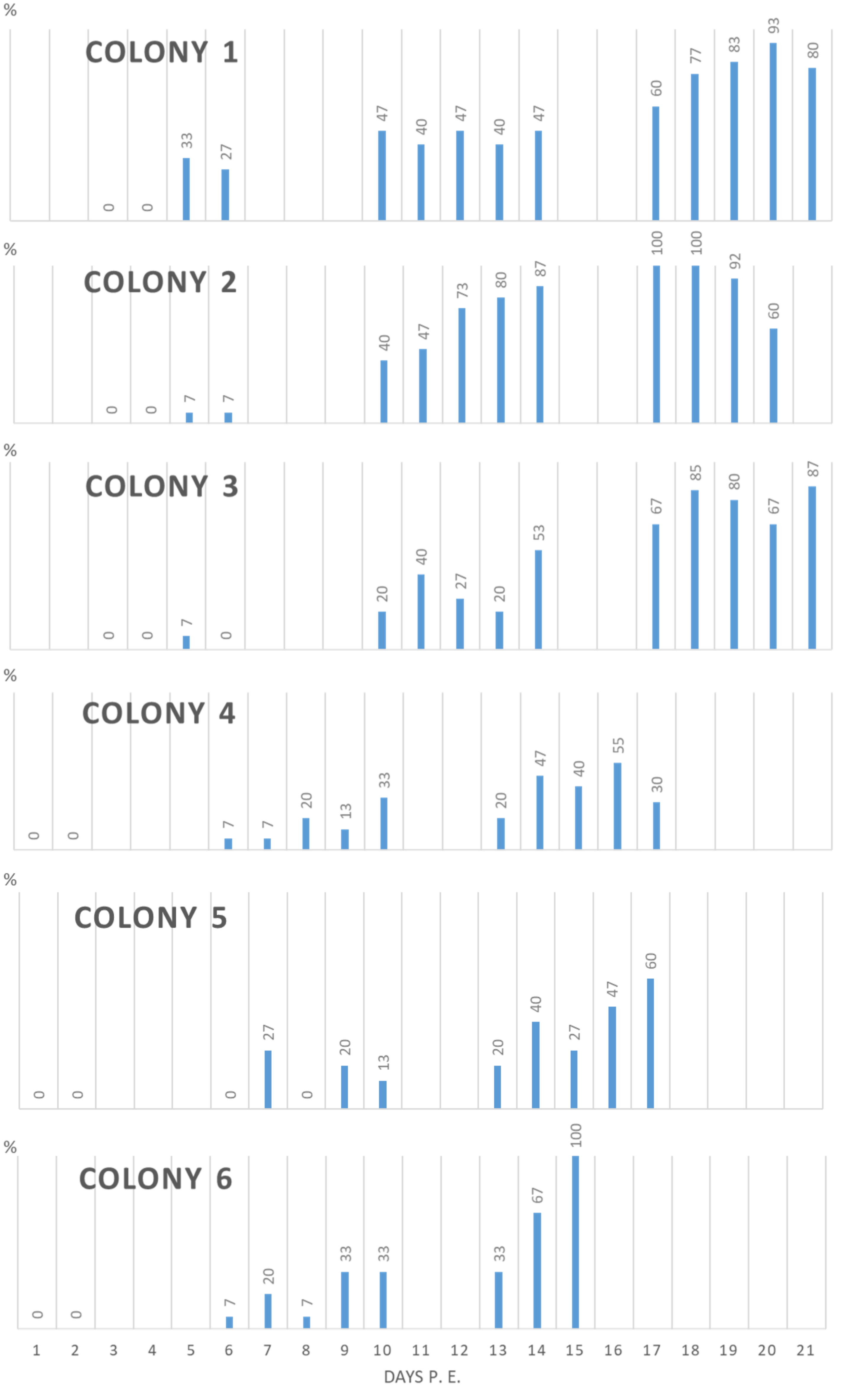

Supplementary Figure 2: Autumn experiment. Two groups of three colonies each were sampled from day 1 post emergence (p.e) until day 22 p.e. The graphs show the percentage of bees infected by *N. ceranae* per day of sampling.

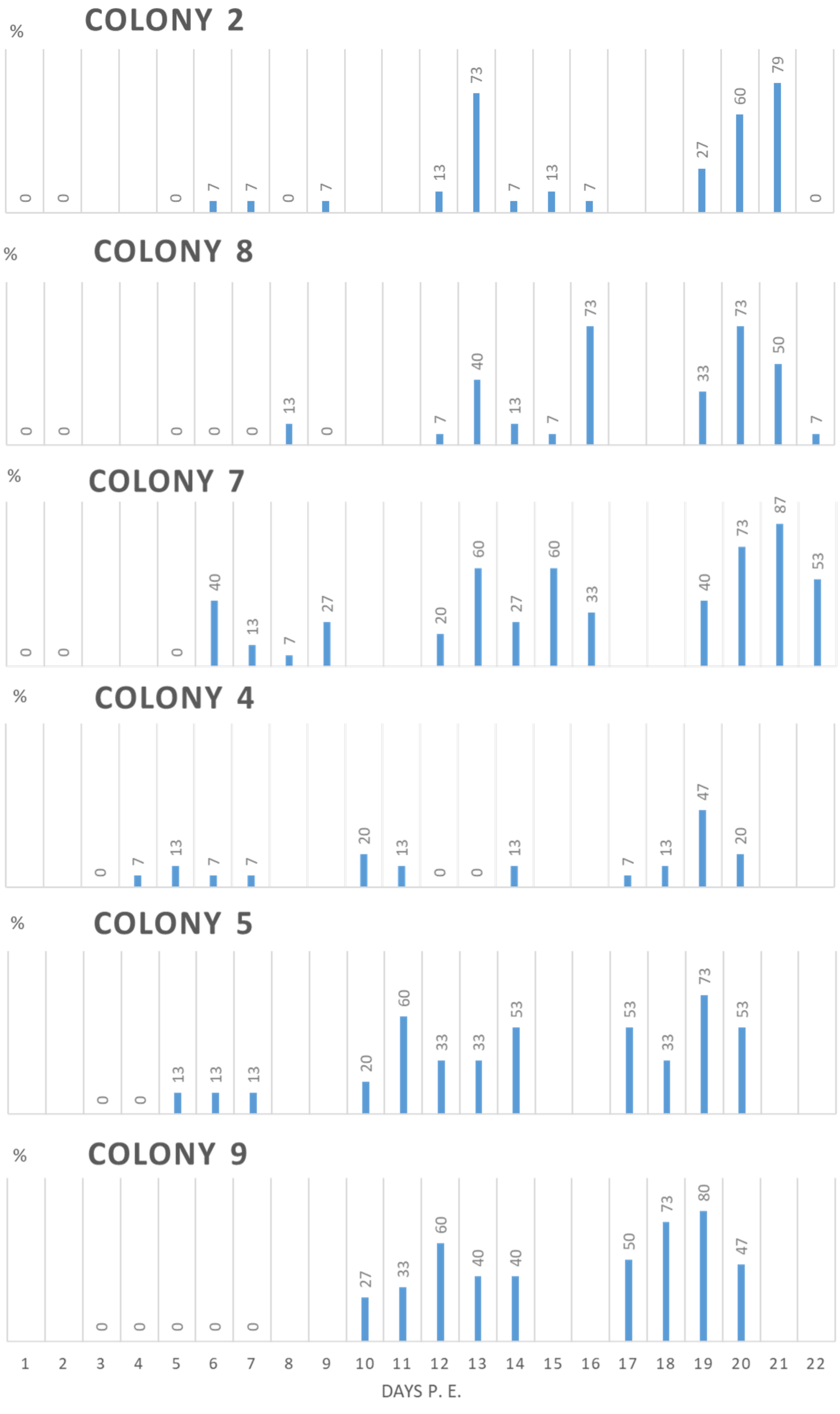

Supplement: Supplementary file 1 [file DataSheet_1.zip › Figure S2.pdf]
