# Supplementary material for: Age and Season Effect the Timing of Adult Worker Honeybee Infection by Nosema ceranae
Source: Front Cell Infect Microbiol. 2022 Jan 28;11:823050. doi: 10.3389/fcimb.2021.823050 (PMC8836290; doi:10.3389/fcimb.2021.823050)
Supplement: Supplementary file 1 [file DataSheet_1.zip › Table S1.pdf]

Supplementary Table 1. Results of the best fitting model for the probability of *N. ceranae* infection. Fixed effects of the generalized linear mixed model with coefficients for each variable, SE, z-value and p-value.

	**Coeffs.**	**Std. Error**	**z value**	**p value**
Intercept	- 2.03	0.25	- 8.17	< 0.001
Age	73.38	7.85	9.35	< 0.001
Age^2	- 20.75	6.66	- 3.12	0.002
Season (spring)	1.22	0.25	4.935.93	< 0.001
Age * Season	25.21	10.78	2.33	0.019
Age^2 * Season	8.87	9.94	0.94	0.347
