# Supplementary material for: Age and Season Effect the Timing of Adult Worker Honeybee Infection by Nosema ceranae
Source: Front Cell Infect Microbiol. 2022 Jan 28;11:823050. doi: 10.3389/fcimb.2021.823050 (PMC8836290; doi:10.3389/fcimb.2021.823050)
Supplement: Supplementary file 1 [file DataSheet_1.zip › Table S2.pdf]

**Supplementary Table 2.** Results of the best fitting model for the *N. ceranae-PTP3* load in infected bees. Fixed effects of the linear mixed model with coefficients for each variable, SE and t-value.

| | **Coeffs.** | **Std. Error** | **t value** |
|---|---|---|---|
| Intercept | - 6.40 | 0.84 | - 7.64 |
| Age | 0.11 | 0.05 | 2.14 |
| Season (spring) | - 2.07 | 1.09 | - 1.88 |
| Age * Season | 0.21 | 0.07 | 2.99 |
