# Supplementary figures and images for: Age and Season Effect the Timing of Adult Worker Honeybee Infection by Nosema ceranae
Source: Front Cell Infect Microbiol. 2022 Jan 28;11:823050. doi: 10.3389/fcimb.2021.823050 (PMC8836290; doi:10.3389/fcimb.2021.823050)

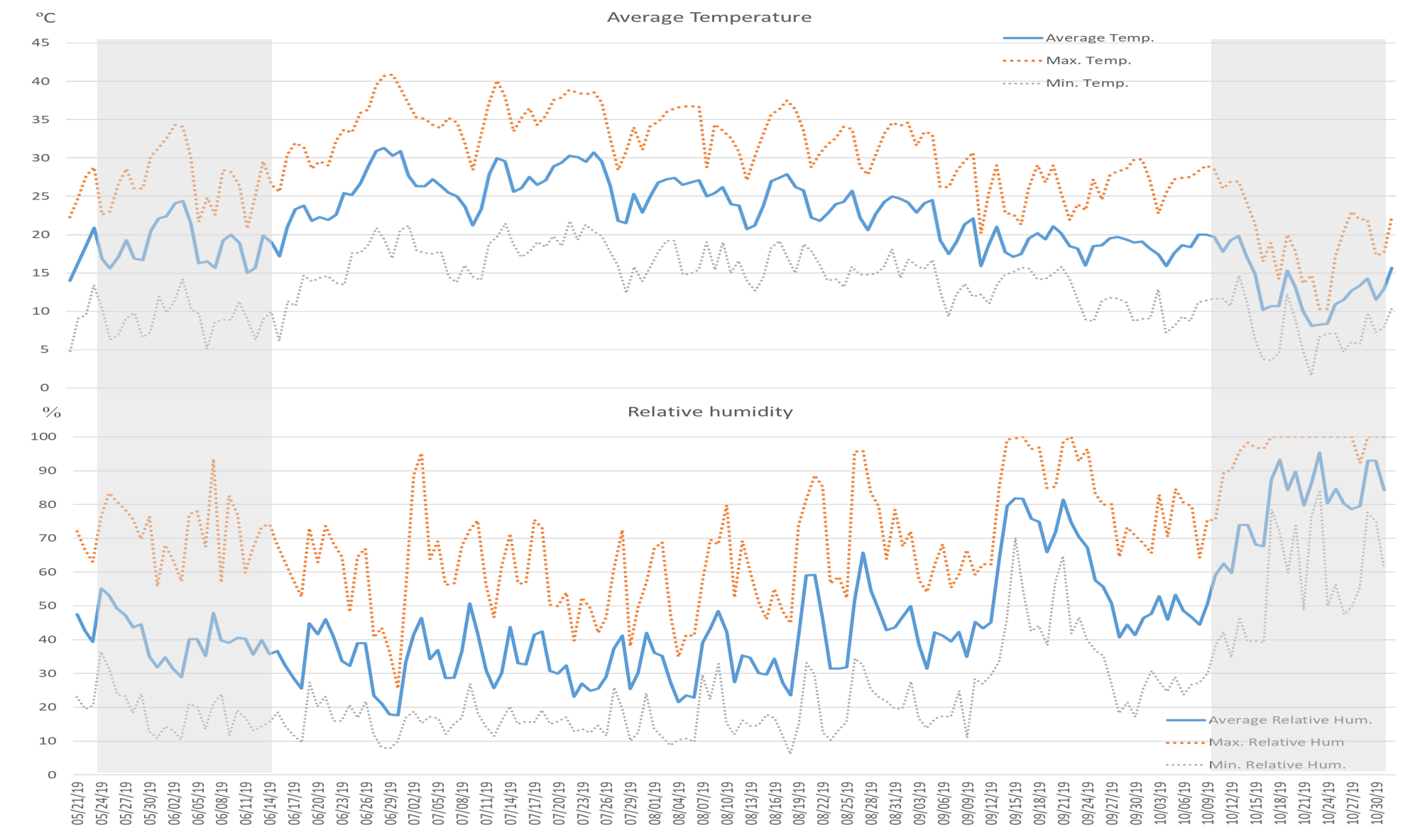

Supplement: Supplementary file 1 [file DataSheet_1.zip › Figure S1.tif]
